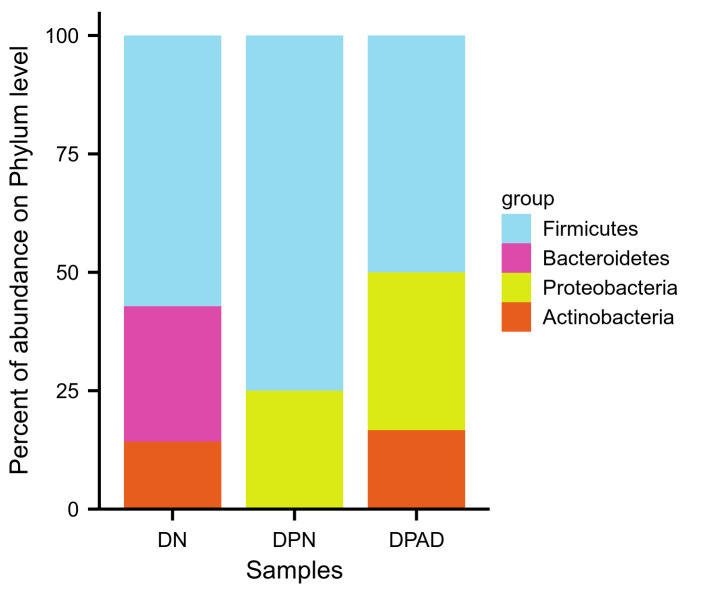# Gut microbiota and diabetic neuropathy/peripheral artery disease: A two-sample Mendelian randomization study investigating risk factors for diabetic foot ulcers

**DOI:** 10.1097/MD.0000000000043637

**Published:** 2025-08-22

**Authors:** Guangjun Tang, Ying Wang, Junde Wu, Ruizhen Zhu, Hui Guo, Yunhui Zhang, Zhaojun Chen

**Affiliations:** a Beijing University of Chinese Medicine, Beijing, China; b Beijing University of Chinese Medicine Third Affiliated Hospital, Beijing, China.

**Keywords:** causal relationship, diabetic foot ulcers, gut microbiota, Mendelian randomization

## Abstract

Diabetic foot ulcers (DFUs) represent a significant complication affecting the lower extremities in individuals with diabetes. The development and progression of DFUs are primarily influenced by diabetic neuropathy (DN) and diabetic peripheral artery disease (DPAD). Recent studies indicate a novel link between the gut microbiota and these risk factors. However, it remains unclear whether a causal relationship exists between them. We obtained data on gut microbiota, derived from publicly available genome-wide association studies. Additionally, we included data on DN, diabetic polyneuropathy (DPN), and DPAD from the FinnGen consortium, considering these conditions as primary risk factors for the onset of DFUs. We employed the inverse variance weighting method for the primary effect analysis and conducted 3 sensitivity tests to ensure the robustness of the results. Our analysis revealed that 7 genera are associated with DN, 8 are associated with DPN, and 12 are associated with DPAD. Notably, the Lachnospiraceae emerged as a common risk factor for both DN [OR = 1.392, 95% CI (1.031, 1.880), *P* = .031] and DPAD [OR = 1.152, 95% CI (1.019, 1.303), *P* = .024]. Conversely, the *Acidaminococcaceae* was identified as a shared protective factor against both DPN [OR = 0.620, 95% CI (0.460, 0.837), *P* = .002] and DPAD [OR = 0.814, 95% CI (0.691, 0.959), *P* = .014]. The sensitivity analysis indicated minimal evidence of bias in this study. Our research findings suggest a potential causal relationship between gut microbiota and the key risk factors for developing DFUs. This suggests that early detection and prevention of this serious diabetic complication might be achievable through gut microbiota modulation.

## 1. Introduction

Diabetic foot ulcers (DFUs) represent a serious lower extremity complication of diabetes, impacting around 18.6 million individuals globally and 1.6 million people annually in the United States.^[1]^ The lifetime risk of developing a DFUs ranges from 19% to 34%. Once a DFU occurs, the prognosis is grim, with a mortality rate of 50% to 70% within 5 years. Additionally, the recurrence rate within 3 to 5 years is 65%, and the likelihood of a lifetime lower limb amputation is 20%.^[2]^ Additionally, DFUs require prolonged treatment and are costly to manage, imposing significant mental and financial strain on patients.^[3]^ DFUs typically result from diabetic neuropathy (DN) and/or diabetic peripheral artery disease (DPAD).^[4]^ It is conservatively estimated that at least 50% of adults diagnosed with diabetes have encountered neuropathy at some stage in their lives. Any form of neuropathy increases the risk of DFUs by approximately 7 times.^[5]^ Moreover, impaired pain perception caused by associated DN often leads to delayed identification and diagnosis of DFUs.^[6]^ The lifetime prevalence of DPAD ranges from 20% to 50%^[7]^ and serves as a significant contributing factor in 50% to 70% of DFU cases. The occurrence of DPAD increases the risk of delayed wound healing, infections, amputations, and even mortality,^[8]^ significantly jeopardizing the health of diabetic patients. DPAD is characterized by elevated blood glucose, excessive insulin levels, and abnormal blood lipids, which collectively contribute to elevated systemic inflammation and oxidative stress.^[9]^ Among diabetic patients, diminished blood flow due to the accumulation of lipid plaques in blood vessels can worsen the progression of complications such as ulcers. This can result in delayed healing, increased inflammation, and the development of gangrene,^[10,11]^ ultimately contributing to elevated rates of amputations.^[12]^ Recent studies have indicated^[13,14]^ that effectively controlling the progression of DN and DPAD is crucial for the early prevention and treatment of DFUs. Yet, early intervention strategies focusing on nerve and peripheral artery lesions in DFUs have shown limited effectiveness. Hence, there is a need to investigate preventive and management approaches focused on DN and DPAD in high-risk diabetic feet. This effort aims to decrease the incidence of DFUs and lower the risk of lower limb amputations.

The gut microbiota consists of microorganisms residing in the human intestine. Often described as the “second genome” or the “second brain” of the body, it plays a pivotal role in numerous physiological functions. An altered or abnormal state of the gut microbiota is associated with the development of several common metabolic conditions. These include obesity, diabetes and its complications, nonalcoholic fatty liver disease, metabolic heart disease, and malnutrition.^[15]^ Recent studies and experiments^[16]^ have shown that DFU patients exhibit a more disturbed distribution of gut microbiota. This disrupted gut microbiota distribution can accelerate or induce the occurrence and development of DFUs. Additionally, the gut microbiota has been found to play a role in regulating patients with symmetrical polyneuropathy in diabetes.^[17]^ It is considered a significant factor in interventions for DPAD.^[18]^ However, insufficient research is currently available to establish a clear causal relationship between the gut microbiota and these risk factors associated with DFUs.

Mendelian randomization (MR) is a well-established research method that enables the inference of causal relationships from data collected in genome-wide association studies. Utilizing MR can effectively reduce confounding and reverse causality biases, allowing for precise analysis of the cause-and-effect relationship between exposure factors and outcomes.^[19]^ In fact, MR has been shown to be an effective tool in evaluating the causal link between gut microbiota and various diseases.^[20]^ To investigate the causal relationship between gut microbiota and the major risk factors associated with DFUs, we utilized MR analysis in this study.

## 2. Materials and methods

### 2.1. MR design

**Figure 1.**
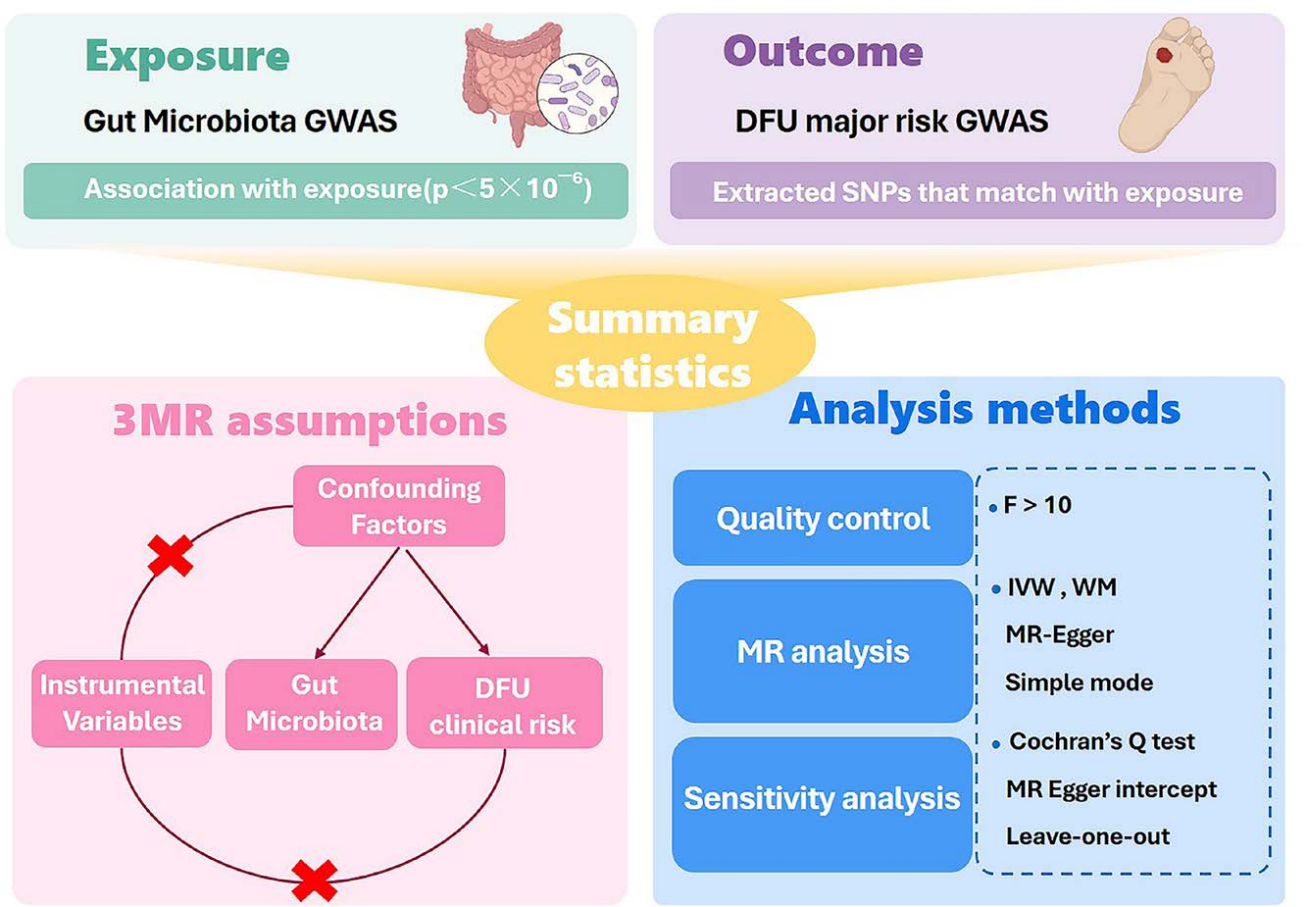
A visual representation of study design. DFU = diabetic foot ulcer, IVW = inverse variance weighted, MR = mendelian randomization.

To investigate the potential causal relationship between gut microbiota and clinical risk factors associated with DFUs, we utilized a two-sample MR approach in this study. For this purpose, relevant single nucleotide polymorphisms (SNPs) were used as instrumental variables (IVs). Three assumptions were required to be met: ① a strong association between the IVs and exposure; ② no correlation between the IVs and any confounding variables; and ③ the effect of the IVs on the outcome is solely mediated by the exposure (a detailed flowchart of the study design is shown in Fig. 1).

### 2.2. Data sources and study population

The gut microbiota data used in this study was collected from the MiBioGen consortium. This data was derived from an ethnic genome-wide association study (GWAS) analysis conducted on a collective sample size of 34,024 individuals across 18 diverse cohorts.^[21]^ The major risk factors for DFUs were identified as DN, diabetic polyneuropathy (DPN), and DPAD, sourced from data available through the FinnGen consortium (https://www.finngen.fi/en/, Table 1).

**Table 1:** The GWAS datasets for outcome.

GWAS-ID	Disease	Sample	Case	Control	Population
finngen_R9_DM_NEUROPATHY	DN	274,660	2843	217,817	European
finngen_R9_DM_POLYNEURO	DPN	375,482	1048	374,434	European
finngen_R7_DM_PERIPHATHERO	DPAD	236,794	11197	225,597	European

GWAS = genome-wide association studies, DN = diabetic neuropathy, DPAD = diabetic peripheral artery disease, DPN = diabetic polyneuropathy.

### 2.3. Instrumental variable selection

We narrowed our focus within the gut microbiota dataset to 211 bacterial classifications ranging from the phylum to the genus level. However, any unidentified or ambiguous classifications were omitted, leading to the inclusion of 117 bacterial genera for our MR analysis. To identify SNPs associated with exposure in the gut microbiota GWAS dataset, we utilized a significance threshold of *P*-value (*P* < 5.0 × 10^‐6^) and also ensured that the linkage disequilibrium (LD) coefficient *r*^2^ < 0.001.^[22]^ Furthermore, we mitigated the potential impact of LD within a region spanning 10,000 kb to uphold the independence between the SNPs. Subsequently, we extracted SNP information associated with both exposures and compared the results with effector allele pairs to achieve precise dataset matching. The strength of exposure to genetic tools was ensured by calculating *F*-statistics and excluding SNPs with *F*-statistics <10.^[23]^ The *F*-statistics is as follows: *R*^2^ (N − *K* − 1)/[*K*(1 − *R*^2^)], where *R*^2^ denotes the explained variance of IV exposure, N represents the effective sample size, and *K* stands for the number of variants included in the IV model.

### 2.4. Statistical methods and sensitivity analysis

In this study, we performed a thorough analysis to investigate the potential causal relationship between gut microbiota and risk factors for DFUs. We employed 4 different analytical methods: inverse variance weighted (IVW), MR-Egger, weighted median (WM), and simple mode, to ensure a comprehensive examination of the data. As the IVW demonstrated higher test efficacy compared to the other 3 methods, it was selected as the primary method of analysis for validating the results in this study^[24]^; *P* < .05 was considered the threshold of significance. The leave-one-out analysis was employed to investigate whether individual SNPs were driving causal associations by systematically excluding each instrumental variable one at a time. The MR-Egger intercept was utilized to assess the multivariate validity of the relationship between the instrumental variable and other potential confounders, ensuring that the selected instrumental variables did not influence outcome variables through pathways other than exposure factors. In this study, the detection of a statistically significant result (*P* < .05) in the MR-Egger intercept analysis implies the existence of horizontal pleiotropy. Additionally, to assess heterogeneity, Cochran *Q* test was employed, and a statistically significant result (*P* < .05) would indicate significant heterogeneity within the analyses.^[25]^ We conducted all statistical analyses in this study using R version 4.2.3 and RStudio software. Specifically, we employed a two-sample MR package, which is compatible with both software platforms.

## 3. Results

At the phylum level, there are 4 potential causal factors associated with the risk of developing diabetic foot ulcers. Among these, Firmicutes has the highest proportion, accounting for 57.14%, 75%, and 50% in the DN, DPN, and DPAD groups, respectively. *Actinobacteria* accounts for 14.29% and 16.67% in the DN and DPAD groups, respectively. Proteobacteria accounts for 25% and 33.33% in the DPN and DPAD groups, while Bacteroidetes accounts for 28.57% in the DN group (Figure S1, Supplemental Digital Content, https://links.lww.com/MD/P620).

### 3.1. Causal effects of gut microbiota on DN

**Figure 2.**
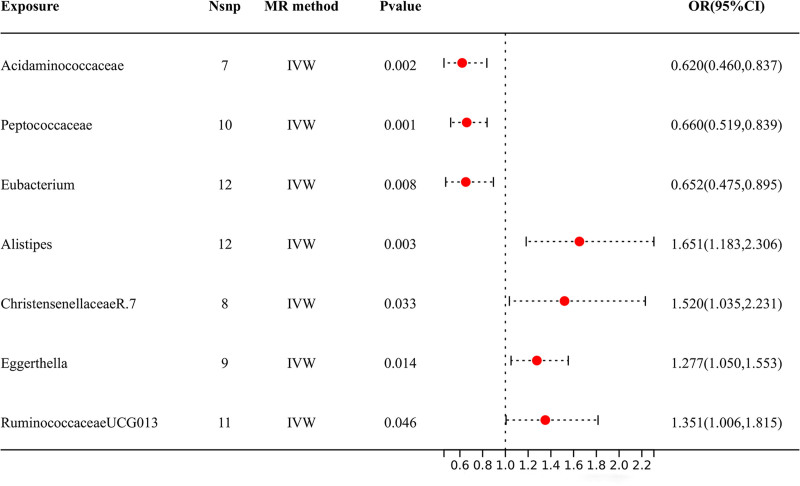
The IVW analysis results of the effect of GM on DN. DN = diabetic neuropathy, GM = gut microbiota, IVW = inverse variance weighted, OR = odds ratio.

According to the results shown in Figure 2, 7 specific genera of gut microbiota were found to have a causal relationship with DN. The IVW analysis indicated that *Peptococcaceae* [OR = 0.660, 95% CI (0.519, 0.839), *P* = .001], *Acidaminococcaceae* [OR = 0.620, 95% CI (0.460, 0.837), *P* = .002], and *Eubacterium coprostanoligene* [OR = 0.652, 95% CI (0.475, 0.895), *P* = .008] had suggestive protective effects against DN. Conversely, *Eggerthella* [OR = 1.277, 95% CI (1.050, 1.553), *P* = .014], *ChristensenellacaeR.7* [OR = 1.520, 95% CI (1.035, 2.231), *P* = .033], *Ruminococcaceae* [OR = 1.351, 95% CI (1.006, 1.815), *P* = .046], and *Alistipes* [OR = 1.651, 95% CI (1.183, 2.306), *P* = .003] were identified as risk factors for DN. The dependable direction and effect size of the estimates obtained from additional MR models, such as WM, MR-Egger, and simple mode, strengthened the causal inference (Table S1, Supplemental Digital Content, https://links.lww.com/MD/P621).

### 3.2. Causal effects of gut microbiota on DPN

**Figure 3.**
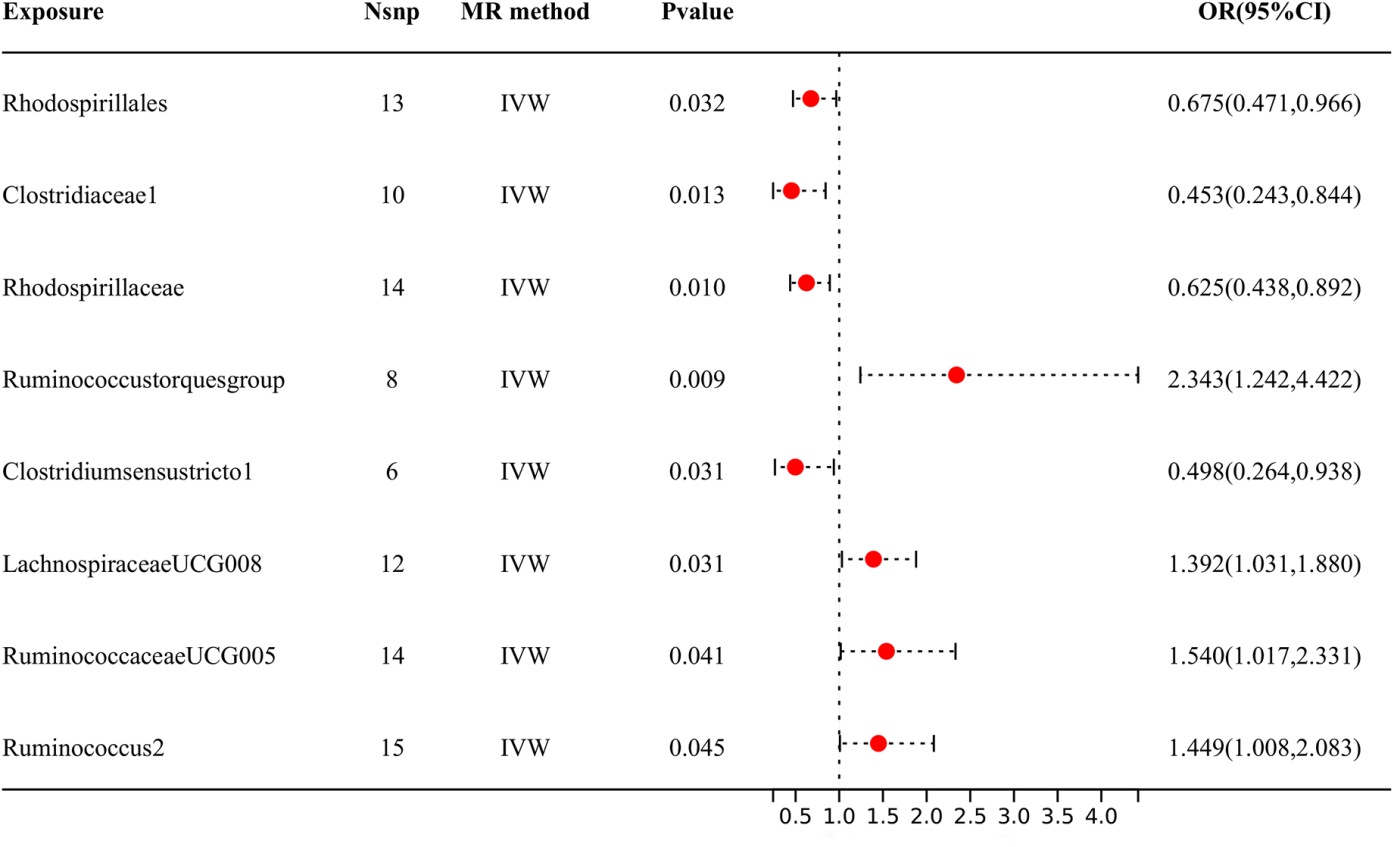
The IVW analysis results of the effect of GM on DPN. DPN = diabetic polyneuropathy, GM = gut microbiota, IVW = inverse variance weighted, OR = odds ratio.

According to the results shown in Figure 3, 8 specific genera of gut microbiota were found to have a causal relationship with DPN. The IVW analysis indicated that *Clostridiaceae1* [OR = 0.453, 95% CI (0.243, 0.844), *P* = .013], *Rhodospirillaceae* [OR = 0.625, 95% CI (0.438, 0.892), *P* = .010], *Clostridium sensustricto1* [OR = 0.498, 95% CI (0.264, 0.938), *P* = .031], and *Rhodospirillales* [OR = 0.675, 95% CI (0.471, 0.966), *P* = .032] had suggestive protective effects against DN. Conversely, *Ruminococcus2* [OR = 1.449, 95% CI (1.008, 2.083), *P* = .045], *RuminococcaceaeUCG005* [OR = 1.540, 95% CI (1.017, 2.331), *P* = .041], *Ruminococcus torques group* [OR = 2.343, 95% CI (1.242, 4.422), *P* = .009], and *LachnospiraceaeUCG008* [OR = 1.392, 95% CI (1.031, 1.880), *P* = .031] were identified as risk factors for DPN. The dependable direction and effect size of the estimates obtained from additional MR models, such as WM, MR-Egger, and simple mode, strengthened the causal inference (Table S1, Supplemental Digital Content, https://links.lww.com/MD/P621).

### 3.3. Causal effects of gut microbiota on DPAD

**Figure 4.**
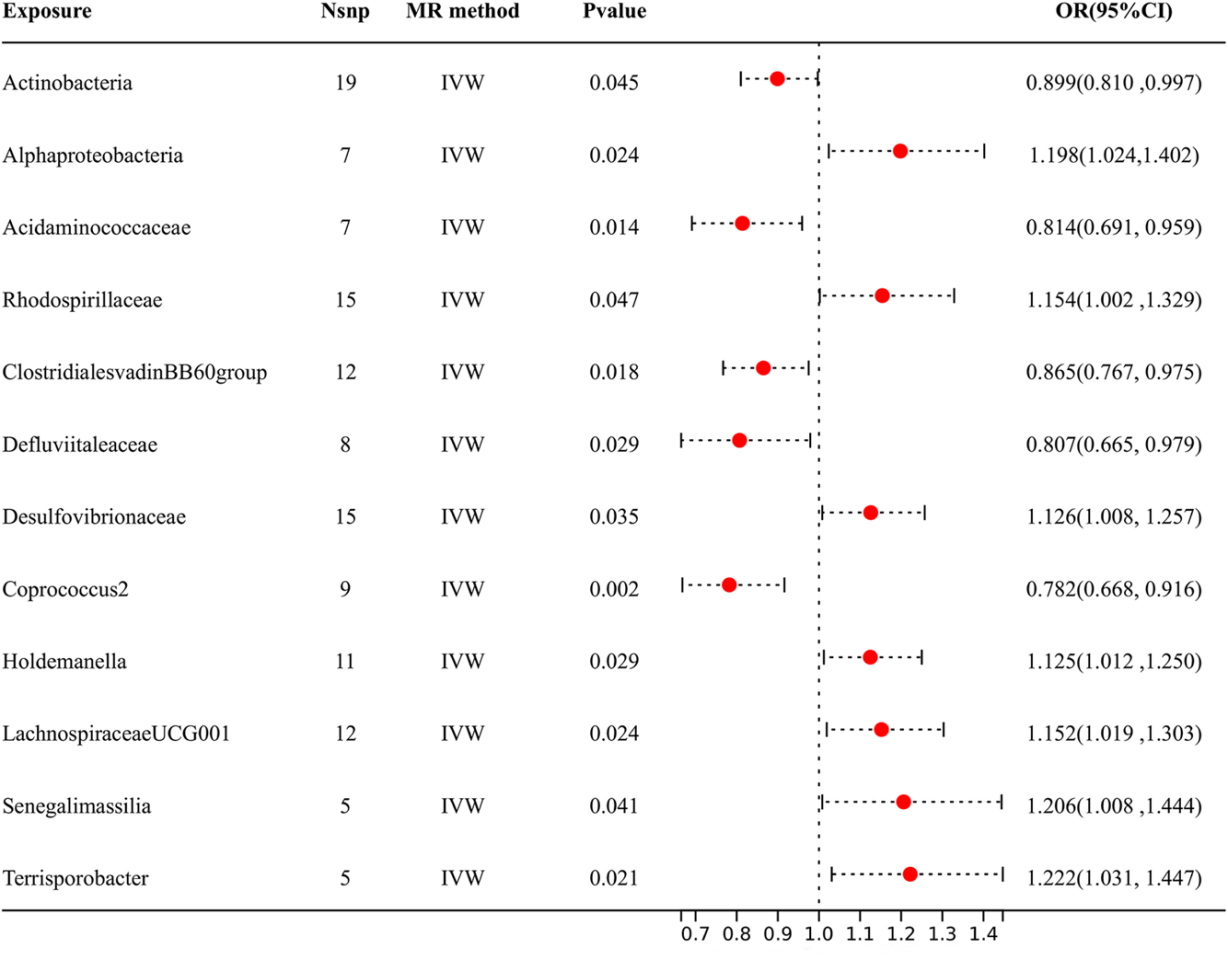
The IVW analysis results of the effect of GM on DPAD. DPAD = diabetic peripheral artery disease, GM = gut microbiota, IVW = inverse variance weighted, OR = odds ratio.

According to the results shown in Figure 4, 12 specific genera of gut microbiota were found to have a causal relationship with DPAD. The IVW analysis indicated that *Actinobacteria* [OR = 0.899, 95% CI (0.810, 0.997), *P* = .045], *Desulfovibrionaceae* [OR = 0.807, 95% CI (0.665, 0.979), *P* = .029], *Defluviitaleaceae* [OR = 0.856, 95% CI (0.752, 0.975), *P* = .019], *Acidaminococcaceae* [OR = 0.814, 95% CI (0.691, 0.959, *P* = .014], *Coprococcus2* [OR = 0.790, 95% CI (0.665, 0.938), *P* = .007], *ClostridialesvadinBB60* [OR = 0.865, 95% CI (0.767, 0.975), *P* = .018] and *LachnospiraceaeUCG001* [OR = 1.152, 95% CI (1.019, 1.303), *P* = .024] had suggestive protective effects against DPAD. Conversely, *Alphaproteobacteria* [OR = 1.198, 95% CI (1.024, 1.402), *P* = .024], *Senegalimassilia* [OR = 1.206, 95% CI (1.008, 1.444), *P* = .041], *Rhodospirillaceae* [OR = 1.154, 95% CI (1.002, 1.329), *P* = .047], *Terrisporobacter* [OR = 1.222, 95% CI (1.031, 1.447), *P* = .021] and *Holdemanella* [OR = 1.125, 95% CI (1.012, 1.250), *P* = .029] were identified as risk factors for DPAD. The dependable direction and effect size of the estimates obtained from additional MR models, such as WM, MR-Egger, and simple mode, strengthened the causal inference (Table S1, Supplemental Digital Content, https://links.lww.com/MD/P621).

### 3.4. Sensitivity analysis

**Figure 5.**
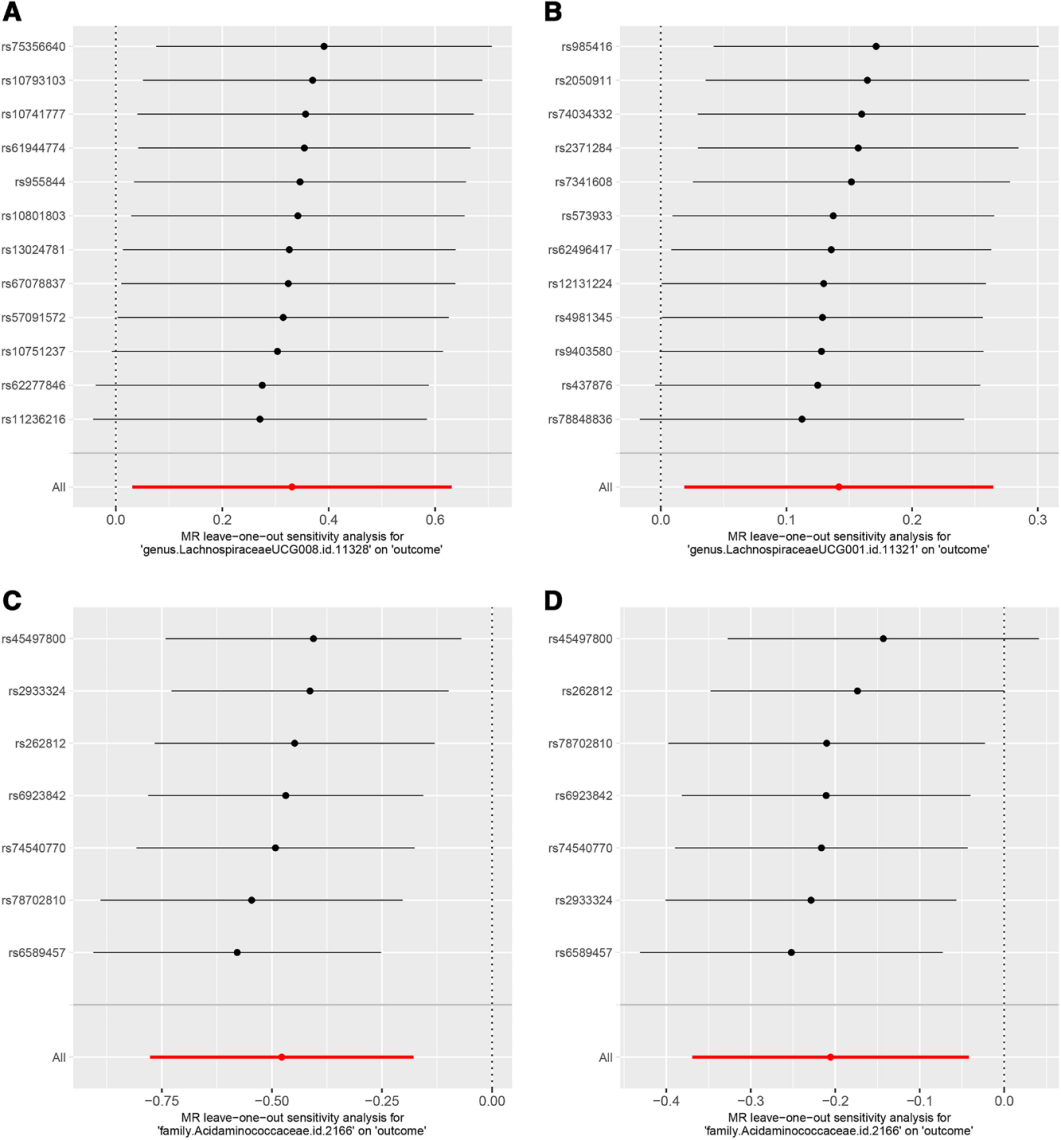
Leave-one-out sensitivity based on IVW model for the shared gut microbiota on DN, DPN, and DPAD. (A) *genus.LachnospiraceaeUCG008* on DPN, (B) *genus.LachnospiraceaeUCG001* on DPAD, (C) *family.Acidaminococcaceae* on DN, (D) *family.Acidaminococcaceae* on DPAD. DN = diabetic neuropathy, DPAD = diabetic peripheral artery disease, DPN = diabetic polyneuropathy, IVW = inverse variance weighted.

Our analysis using the MR-Egger regression intercept method showed no evidence of pleiotropy in the levels of gut microbiota associated with DFUs (*P* > .05, Table 2). Furthermore, Cochran *Q* test results indicated no significant heterogeneity (*P* > .05, Table 2). Additionally, the leave-one-out sensitivity test underscored the robustness of our results, as the removal of any single SNP did not affect the overall findings (Fig. 5).

**Table 2:** The sensitivity analysis results of the effect of GM on DN, DPN, and DPAD.

GM taxa	Heterogeneity test				Pleiotropy test	
	IVW		MR-Egger		MR-Egger	
	*Q*	*P*	*Q*	*P*	Intercept	*P*
*Acidaminococcaceae*	4.943	.551	2.026	.846	‐0.078	.148
*Peptococcaceae*	6.479	.691	2.026	.598	0.006	.853
*Eubacterium coprostanoligene*	11.294	.419	6.442	.339	0.008	.838
*Alistipes*	8.556	.663	11.245	.636	0.039	.444
*ChristensenellaceaeR.7*	7.031	.426	7.922	.330	‐0.019	.746
*Eggerthella*	7.304	.504	6.899	.441	‐0.032	.536
*RuminococcaceaeUCG013*	5.606	.847	6.880	.818	‐0.022	.534
*Rhodospirillales*	15.219	.230	12.719	.312	‐0.100	.169
*Clostridiaceae1*	15.883	.069	13.660	.091	0.077	.287
*Rhodospirillaceae*	16.876	.205	13.280	.349	‐0.123	.097
*Ruminococcustorquesgroup*	2.097	.954	1.974	.922	‐0.023	.738
*Clostridiumsensustricto1*	7.659	.176	7.404	.116	0.035	.730
*LachnospiraceaeUCG008*	5.982	.875	4.089	.943	0.107	.199
*RuminococcaceaeUCG005*	14.623	.331	10.366	.584	‐0.094	.061
*Ruminococcus2*	11.209	.670	10.944	.615	‐0.018	.615
*Actinobacteria*	11.369	.878	10.773	.868	‐0.007	.451
*Alphaproteobacteria*	3.876	.693	2.414	.789	0.033	.281
*Acidaminococcaceae*	4.860	.562	4.654	.459	‐0.011	.669
*Rhodospirillaceae*	21.799	.083	20.758	.078	0.015	.434
*ClostridialesvadinBB60group*	11.110	.434	9.797	.459	0.024	.278
*Defluviitaleaceae*	6.532	.479	6.191	.402	‐0.018	.586
*Desulfovibrionaceae*	16.252	.298	16.244	.236	0.002	.938
*Coprococcus2*	6.718	.567	6.597	.472	0.011	.739
*Holdemanella*	6.049	.811	5.223	.814	‐0.015	.387
*LachnospiraceaeUCG001*	8.994	.622	8.949	.537	‐0.005	.837
*Senegalimassilia*	1.411	.842	1.391	.708	0.005	.896
*Terrisporobacter*	1.525	.822	1.066	.785	‐0.017	.547

GM = gut microbiota, DN = diabetic neuropathy, DPAD = diabetic peripheral artery disease, DPN = diabetic polyneuropathy, IVW = inverse variance weighting.

## 4. Discussion

In this MR study, we discovered that 7 genera were associated with DN, 8 were associated with DPN, and 12 were associated with DPAD. Notably, Lachnospiraceae emerged as a shared risk factor for both DPN and DPAD, while *Acidaminococcaceae* was identified as a shared protective factor for both DN and DPAD. These findings hold positive implications for public health interventions targeted at lowering the risk of developing DFUs.

The evidence linking gut microbiota to DFUs remains limited, with research in this area relatively scarce. However, we propose that there may be 2 main relationships between gut microbiota and DFUs: First, gut microbiota can modulate diabetic neuropathy, a major contributor to the occurrence of DFUs.^[5]^ Diabetic neuropathy primarily encompasses peripheral and autonomic dysfunctions, manifesting symptoms such as pain and sensory abnormalities, and occasionally presenting asymptomatically. These neuropathic conditions frequently precipitate the development of DFUs. Although the exact correlation between the gut microbiota and DN remains uncertain, recent studies suggest a significant association between DN and alterations in gut microbial diversity, including an increase in pathogens. Notably, patients with DN exhibit distinct gut microbiota profiles compared to other groups, characterized by an increase in Firmicutes and *Actinobacteria*, and a decrease in Bacteroidetes. These microbiotic shifts are hypothesized to be linked to insulin resistance.^[26]^ Recent investigations have focused on elucidating the involvement of gut microbiota in neurological diseases, notably chronic pain.^[27]^ Research indicates that bacteria, through their constituent elements and byproducts,^[28,29]^ including alpha-hemolysin (a toxin produced by *Staphylococcus aureus*), can activate nociceptors directly, leading to the induction of spontaneous pain^[30]^ Wang et al^[31]^ noted a significant disruption in the gut microbiota among individuals with DPN. The patients demonstrated a notable decrease in the presence of Bacteroides and Faecalibacterium, along with an increased abundance of *Ruminococcus* torques. These findings are consistent with our research results. Additionally, it was discovered that changes in the abundance of Parabacteroides and levels of tauroursodeoxycholic acid may impact insulin resistance and dyslipidemia, which can ultimately influence the development of DPN. Tanase et al^[32]^ found in a rodent model that high-fat diet-induced ecological disruption was associated with an increased presence of Allobaculum, Lactobacillus, and Bifidobacteria. In a rat model of DPN, Huang et al^[33]^ discovered that abnormalities in microorganisms belonging to the Firmicutes, *Ruminococcaceae*, Bacteroidia, and *Actinobacteria* families may contribute to cognitive dysfunction in DPN patients. Yang et al^[17]^ found that gut microbiota derived from diabetic patients with distal symmetric polyneuropathy (DSPN) could induce a more severe phenotype of peripheral nerve damage in db/db mice. Subsequently, they discovered a distinct composition of gut microbiota in DSPN patients in a randomized controlled trial, characterized by a reduction in potentially beneficial bacteria and an increase in potentially pathogenic bacteria. By modulating the composition and function of gut microbiota, they were able to alleviate the neuropathic symptoms in patients with DSPN, regardless of blood glucose regulation. This highlights the potential therapeutic impact of gut microbiota in DN. While certain characteristics of gut microbiota in individuals with DN have been identified, further exploration is necessary to fully comprehend the impact of gut microbiota on the onset and progression of DN. At present, there is a scarcity of drug interventions accessible for managing DN and its consequent decline in quality of life. Therefore, additional studies are warranted to identify probiotics or prebiotic dietary supplements that can be used to prevent, control, or even treat DN.

Second, gut microbiota can influence DPAD. The disease is defined by diminished blood flow resulting from vasoconstriction or blockage of blood vessels in the lower extremities, affecting more than half of patients with DFUs and hindering the process of ulcer healing.^[34]^ A study has shown that the production of short-chain fatty acids (SCFAs) by gut microbiota is relevant to the progression of atherosclerosis.^[35]^ To examine the link between fecal SCFA levels and atherosclerosis in DPAD patients, Muradi A et al^[36]^ conducted a study with a sample size of 53 individuals diagnosed with DPAD. Through the utilization of mass spectrometry to assess gut flora metabolites in stool samples obtained from DPAD patients who had consented to participate in the study, the investigators obtained compelling findings that established a noteworthy affirmative association amid various markers, including plaque and randomly assessed blood glucose levels. The findings confirmed the hypothesis that elevated SCFA levels, which impede the inflammatory response in adipose tissues and affect cholesterol metabolism, increase the likelihood of atherosclerosis among patients with type 2 diabetes mellitus. Huseini et al^[37]^ investigated the potential efficacy of probiotic administration in rat models for mitigating peripheral vascular disease and enhancing wound healing. The rats were administered kefir, and the results demonstrated that the lactic acid produced by the probiotic bacteria inhibited the growth of harmful microorganisms. This inhibition ultimately resulted in improved wound healing. Apart from lactic acid, other components present in kefir, such as polysaccharides, also significantly enhance wound healing by activating the body’s innate immune response. Furthermore, clinical studies have been conducted investigating the therapeutic potential of probiotics in human subjects with DFUs and associated peripheral vascular disease. A study^[38]^ demonstrated that patients with DFUs who underwent probiotic treatment for a duration of 12 weeks experienced a notable reduction in the dimensions of their ulcers, including length, width, and thickness. These findings suggest that incorporating probiotics into a treatment regimen, such as kefir, can benefit wound healing. By inhibiting the growth of pathogenic microorganisms and stimulating the immune response, probiotics show promise in improving the healing process both in animal models and in individuals with DFUs. Although the specific mechanisms are not yet fully understood, some researchers suggest that probiotics improve DFUs by modulating the local immune response, akin to how other lesions in the body may benefit from probiotic treatment.^[39]^ Thus, by enhancing the diversity and abundance of beneficial gut microbiota through probiotic supplementation, improvements in glucose, insulin, lipid metabolism, and incretion levels may be achieved, potentially aiding in the treatment of DPAD.^[40]^ In a randomized, double-blind, controlled clinical trial, Depoortere et al^[41]^ reported that treatment with *Akkermansia muciniphila* resulted in significant improvements in insulin sensitivity, decreased total plasma cholesterol levels, and a reduced inflammatory response among individuals with DPAD. These findings imply that targeted interventions utilizing specific bacterial strains could be beneficial in ameliorating DPAD. Indeed, a number of bacteria with advanced functional attributes, referred to as next-generation probiotics, have been recognized for their potential in treating particular diseases in hosts. Some of the key bacteria that have attracted significant attention in recent years include *A muciniphila*, *Faecalibacterium prausnitzii*, *Roseburia intestinalis*, *Anaerobutyricum hallii*, and *Ruminococcus bromiiare*. Among these, *A muciniphila* has emerged as a notable candidate, recognized for its potential as a diagnostic tool in dietary interventions. It has shown promise in mitigating metabolic syndrome phenotypes and improving both lipid and glucose metabolism.^[42]^ Similarly, *F prausnitzii* has been suggested as a potential biomarker for evaluating gut inflammation and as a strategy for dietary intervention, according to various studies.^[43]^ Overall, investigating these specific bacterial strains offers promising potential for developing targeted interventions for DPAD, providing a hopeful direction for future research.

Our study should be assessed on multiple fronts. First, it offers robust evidence by further enriching the existing literature on the causal relationship between gut microbiota and the primary risk factors for developing DFUs, even without a randomized controlled trial. Second, our major findings suggest that fecal examination may be a viable strategy to identify individuals at higher risk for DFUs. In addition, the current study identified a range of potential causal relationships between gut microbiota and major risk factors for developing DFUs. These findings highlight specific bacterial strains within the gut microbiome that warrant further investigation in future functional research. This study has several limitations. First, our study’s participants were predominantly of European descent, and we had minimal gut microbiota data from other ethnic groups. This limitation could result in biased estimations and impact the generalizability of our findings. Second, the initial study of gut microbiota focused solely on taxonomic classifications at the phylum, class, order, family, and genus levels. This approach did not incorporate GWAS meta-analyses at the species level. Third, although our Mendelian randomization design mitigates confounding from environmental factors, we acknowledge that antidiabetic medications (e.g., metformin, acarbose) may influence gut microbiota composition. However, the lack of drug-stratified summary statistics in current gut microbiota GWAS precludes direct adjustment for medication effects in our analysis. Future studies should address this limitation by replicating findings in cohorts with detailed medication records and employing multi-omics approaches to identify drug-modifiable microbial pathways.

## 5. Conclusion

In conclusion, this study comprehensively evaluated the causal relationship between gut microbiota and key clinical risk factors for developing DFUs and identified several strains of beneficial microbiota. These insights could be leveraged in public health initiatives to lower the risk of DFUs, suggesting that altering gut microbiota might be an effective strategy for preventing and managing DFUs.

## Acknowledgments

The authors would like to extend their heartfelt appreciation to all the volunteers who generously participated in this study. We are also indebted to the MiBioGen consortium for providing us with the GWAS summary statistics on the gut microbiota. Furthermore, we would like to express our gratitude to the participants and investigators of the FinnGen study.

## Author contributions

**Conceptualization:** Zhaojun Chen.

**Data curation:** Guangjun Tang, Junde Wu.

**Methodology:** Ying Wang.

**Resources:** Ruizhen Zhu, Hui Guo, Yunhui Zhang.

**Software:** Junde Wu, Yunhui Zhang.

**Supervision:** Zhaojun Chen.

**Validation:** Hui Guo.

**Visualization:** Ruizhen Zhu.

**Writing – original draft:** Guangjun Tang.

**Writing – review & editing:** Ying Wang.

## Supplementary Material



**Figure:**