# Supplementary material for: Gut microbiota and diabetic neuropathy/peripheral artery disease: A two-sample Mendelian randomization study investigating risk factors for diabetic foot ulcers
Source: Medicine (Baltimore). 2025 Aug 22;104(34):e43637. doi: 10.1097/MD.0000000000043637 (PMC12384795; doi:10.1097/MD.0000000000043637)
Supplement: Supplementary file 1 [file medi-104-e43637-s002.docx]

**Table S1** All results of the four MR analysis methods.

| **Exposure** | **Nsnp** | **MR method** | **Pvalue** | **OR** | **Lower** | **Upper** | **OR(95%CI)** | **P heterogeneity** | **P pleiotropy** |
| --- | --- | --- | --- | --- | --- | --- | --- | --- | --- |
| **Diabetic neuropathy** |  |  |  |  |  |  |  |  |  |
| Acidaminococcaceae | 7 | IVW | 0.002 | 0.620 | 0.460 | 0.837 | 0.620(0.460,0.837) | 0.551 | 0.148 |
| Acidaminococcaceae | 7 | MR Egger | 0.590 | 1.303 | 0.528 | 3.215 | 1.303(0.528,3.215) | 0.846 |  |
| Acidaminococcaceae | 7 | Weighted median | 0.061 | 0.674 | 0.447 | 1.018 | 0.674(0.447,1.018) |  |  |
| Acidaminococcaceae | 7 | Simple mode | 0.138 | 0.542 | 0.269 | 1.093 | 0.542(0.269,1.093) |  |  |
| Peptococcaceae | 10 | IVW | 0.001 | 0.660 | 0.519 | 0.839 | 0.660(0.519,0.839) | 0.691 | 0.853 |
| Peptococcaceae | 10 | MR Egger | 0.197 | 0.621 | 0.320 | 1.206 | 0.621(0.320,1.206) | 0.598 |  |
| Peptococcaceae | 10 | Weighted median | 0.050 | 0.720 | 0.518 | 1.000 | 0.720(0.518,1.000) |  |  |
| Peptococcaceae | 10 | Simple mode | 0.405 | 1.373 | 0.645 | 2.923 | 1.373(0.645,2.923) |  |  |
| Eubacterium | 12 | IVW | 0.008 | 0.652 | 0.475 | 0.895 | 0.652(0.475,0.895) | 0.419 | 0.838 |
| Eubacterium | 12 | MR Egger | 0.399 | 0.574 | 0.166 | 1.977 | 0.574(0.166,1.977) | 0.339 |  |
| Eubacterium | 12 | Weighted median | 0.278 | 0.780 | 0.498 | 1.221 | 0.780(0.498,1.221) |  |  |
| Eubacterium | 12 | Simple mode | 0.663 | 0.854 | 0.428 | 1.704 | 0.854(0.428,1.704) |  |  |
| Alistipes | 12 | IVW | 0.003 | 1.651 | 1.183 | 2.306 | 1.651(1.183,2.306) | 0.663 | 0.444 |
| Alistipes | 12 | MR Egger | 0.878 | 0.881 | 0.181 | 4.288 | 0.881(0.181,4.288) | 0.636 |  |
| Alistipes | 12 | Weighted median | 0.103 | 1.451 | 0.928 | 2.269 | 1.451(0.928,2.269) |  |  |
| Alistipes | 12 | Simple mode | 0.428 | 1.373 | 0.645 | 2.923 | 1.373(0.645,2.923) |  |  |
| ChristensenellaceaeR.7 | 8 | IVW | 0.033 | 1.520 | 1.035 | 2.231 | 1.520(1.035,2.231) | 0.426 | 0.746 |
| ChristensenellaceaeR.7 | 8 | MR Egger | 0.405 | 1.929 | 0.458 | 8.119 | 1.929(0.458,8.119) | 0.330 |  |
| ChristensenellaceaeR.7 | 8 | Weighted median | 0.332 | 1.316 | 0.756 | 2.290 | 1.316(0.756,2.290) |  |  |
| ChristensenellaceaeR.7 | 8 | Simple mode | 0.711 | 1.182 | 0.506 | 2.761 | 1.182(0.506,2.761) |  |  |
| Eggerthella | 9 | IVW | 0.014 | 1.277 | 1.050 | 1.553 | 1.277(1.050,1.553) | 0.504 | 0.536 |
| Eggerthella | 9 | MR Egger | 0.276 | 1.696 | 0.706 | 4.072 | 1.696(0.706,4.072) | 0.441 |  |
| Eggerthella | 9 | Weighted median | 0.163 | 1.216 | 0.924 | 1.599 | 1.216(0.924,1.599) |  |  |
| Eggerthella | 9 | Simple mode | 0.546 | 1.152 | 0.742 | 1.790 | 1.152(0.742,1.790) |  |  |
| RuminococcaceaeUCG013 | 11 | IVW | 0.046 | 1.351 | 1.006 | 1.815 | 1.351(1.006,1.815) | 0.847 | 0.534 |
| RuminococcaceaeUCG013 | 11 | MR Egger | 0.226 | 1.759 | 0.750 | 4.122 | 1.759(0.750,4.122) | 0.818 |  |
| RuminococcaceaeUCG013 | 11 | Weighted median | 0.253 | 1.260 | 0.848 | 1.873 | 1.260(0.848,1.873) |  |  |
| RuminococcaceaeUCG013 | 11 | Simple mode | 0.701 | 1.146 | 0.582 | 2.256 | 1.146(0.582,2.256) |  |  |
| **Diabetic polyneuropathy** |  |  |  |  |  |  |  |  |  |
| Rhodospirillales | 13 | IVW | 0.032 | 0.675 | 0.471 | 0.966 | 0.675(0.471,0.966) | 0.230 | 0.169487 |
| Rhodospirillales | 13 | MR Egger | 0.413 | 1.797 | 0.466 | 6.928 | 1.797(0.466,6.928) | 0.312 |  |
| Rhodospirillales | 13 | Weighted median | 0.658 | 0.900 | 0.565 | 1.434 | 0.900(0.565,1.434) |  |  |
| Rhodospirillales | 13 | Simple mode | 0.862 | 0.927 | 0.404 | 2.130 | 0.927(0.404,2.130) |  |  |
| Clostridiaceae1 | 10 | IVW | 0.013 | 0.453 | 0.243 | 0.844 | 0.453(0.243,0.844) | 0.069 | 0.286841 |
| Clostridiaceae1 | 10 | MR Egger | 0.088 | 0.170 | 0.029 | 1.018 | 0.170(0.029,1.018) | 0.091 |  |
| Clostridiaceae1 | 10 | Weighted median | 0.032 | 0.441 | 0.209 | 0.930 | 0.441(0.209,0.930) |  |  |
| Clostridiaceae1 | 10 | Simple mode | 0.105 | 0.257 | 0.059 | 1.126 | 0.257(0.059,1.126) |  |  |
| Rhodospirillaceae | 14 | IVW | 0.010 | 0.625 | 0.438 | 0.892 | 0.625(0.438,0.892) | 0.205 | 0.096577 |
| Rhodospirillaceae | 14 | MR Egger | 0.308 | 2.079 | 0.541 | 7.992 | 2.079(0.541,7.992) | 0.349 |  |
| Rhodospirillaceae | 14 | Weighted median | 0.064 | 0.647 | 0.408 | 1.026 | 0.647(0.408,1.026) |  |  |
| Rhodospirillaceae | 14 | Simple mode | 0.142 | 0.519 | 0.228 | 1.181 | 0.519(0.228,1.181) |  |  |
| Ruminococcustorquesgroup | 8 | IVW | 0.009 | 2.343 | 1.242 | 4.422 | 2.343(1.242,4.422) | 0.954 | 0.737938 |
| Ruminococcustorquesgroup | 8 | MR Egger | 0.288 | 3.290 | 0.445 | 24.351 | 3.290(0.445,24.531) | 0.922 |  |
| Ruminococcustorquesgroup | 8 | Weighted median | 0.105 | 1.961 | 0.869 | 4.423 | 1.961(0.869,4.423) |  |  |
| Ruminococcustorquesgroup | 8 | Simple mode | 0.366 | 1.837 | 0.536 | 6.302 | 1.837(0.536,6.302) |  |  |
| Clostridiumsensustricto1 | 6 | IVW | 0.031 | 0.498 | 0.264 | 0.938 | 0.498(0.264,0.938) | 0.176 | 0.729506 |
| Clostridiumsensustricto1 | 6 | MR Egger | 0.346 | 0.356 | 0.054 | 2.372 | 0.356(0.054,2.372) | 0.116 |  |
| Clostridiumsensustricto1 | 6 | Weighted median | 0.016 | 0.410 | 0.199 | 0.845 | 0.410(0.199,0.845) |  |  |
| Clostridiumsensustricto1 | 6 | Simple mode | 0.175 | 0.365 | 0.105 | 1.274 | 0.365(0.105,1.274) |  |  |
| LachnospiraceaeUCG008 | 12 | IVW | 0.031 | 1.392 | 1.031 | 1.880 | 1.392(1.031,1.880) | 0.875 | 0.198879 |
| LachnospiraceaeUCG008 | 12 | MR Egger | 0.377 | 0.485 | 0.105 | 2.243 | 0.485(0.105,2.243) | 0.943 |  |
| LachnospiraceaeUCG008 | 12 | Weighted median | 0.350 | 1.215 | 0.807 | 1.829 | 1.215(0.807,1.829) |  |  |
| LachnospiraceaeUCG008 | 12 | Simple mode | 0.626 | 1.188 | 0.605 | 2.333 | 1.188(0.605,2.333) |  |  |
| RuminococcaceaeUCG005 | 14 | IVW | 0.041 | 1.540 | 1.017 | 2.331 | 1.540(1.017,2.331) | 0.331 | 0.061397 |
| RuminococcaceaeUCG005 | 14 | MR Egger | 0.019 | 4.396 | 1.508 | 12.819 | 4.396(1.508,12.819) | 0.584 |  |
| RuminococcaceaeUCG005 | 14 | Weighted median | 0.126 | 1.546 | 0.885 | 2.701 | 1.546(0.885,2.701) |  |  |
| RuminococcaceaeUCG005 | 14 | Simple mode | 0.727 | 1.176 | 0.482 | 2.868 | 1.176(0.482,2.868) |  |  |
| Ruminococcus2 | 15 | IVW | 0.045 | 1.449 | 1.008 | 2.083 | 1.449(1.008,2.083) | 0.670 | 0.615486 |
| Ruminococcus2 | 15 | MR Egger | 0.217 | 1.786 | 0.744 | 4.285 | 1.786(0.744,4.285) | 0.615 |  |
| Ruminococcus2 | 15 | Weighted median | 0.240 | 1.385 | 0.805 | 2.384 | 1.385(0.805,2.384) |  |  |
| Ruminococcus2 | 15 | Simple mode | 0.363 | 1.470 | 0.659 | 3.281 | 1.470(0.659,3.281) |  |  |
| Bacterial taxa（exposure） | Nsnp | MR method | Pvalue | OR | Lower | Upper | OR(95%CI) | P heterogeneity | P pleiotropy |
| **Diabetic peripheral artery disease** |  |  |  |  |  |  |  |  |  |
| Actinobacteria | 19 | IVW | 0.045 | 0.899 | 0.810 | 0.997 | 0.899(0.810 ,0.997) | 0.878 | 0.451 |
| Actinobacteria | 19 | MR Egger | 0.796 | 0.971 | 0.778 | 1.211 | 0.971(0.778 ,1.211) | 0.868 |  |
| Actinobacteria | 19 | Weighted median | 0.165 | 0.897 | 0.770 | 1.046 | 0.897(0.770 ,1.046) |  |  |
| Actinobacteria | 19 | Simple mode | 0.791 | 0.968 | 0.765 | 1.226 | 0.968(0.765,1.226) |  |  |
| Alphaproteobacteria | 7 | IVW | 0.024 | 1.198 | 1.024 | 1.402 | 1.198(1.024,1.402) | 0.693 | 0.281 |
| Alphaproteobacteria | 7 | MR Egger | 0.598 | 0.844 | 0.469 | 1.521 | 0.844(0.469, 1.521) | 0.789 |  |
| Alphaproteobacteria | 7 | Weighted median | 0.189 | 1.154 | 0.932 | 1.430 | 1.154(0.932, 1.430) |  |  |
| Alphaproteobacteria | 7 | Simple mode | 0.503 | 1.106 | 0.838 | 1.461 | 1.106(0.838,1.461) |  |  |
| Acidaminococcaceae | 7 | IVW | 0.014 | 0.814 | 0.691 | 0.959 | 0.814(0.691, 0.959) | 0.562 | 0.669 |
| Acidaminococcaceae | 7 | MR Egger | 0.716 | 0.907 | 0.553 | 1.488 | 0.907(0.553 ,1.488) | 0.459 |  |
| Acidaminococcaceae | 7 | Weighted median | 0.157 | 0.850 | 0.678 | 1.065 | 0.850(0.678, 1.065) |  |  |
| Acidaminococcaceae | 7 | Simple mode | 0.630 | 0.909 | 0.628 | 1.314 | 0.909(0.628,1.314) |  |  |
| Rhodospirillaceae | 15 | IVW | 0.047 | 1.154 | 1.002 | 1.329 | 1.154(1.002 ,1.329) | 0.083 | 0.434 |
| Rhodospirillaceae | 15 | MR Egger | 0.974 | 0.993 | 0.672 | 1.468 | 0.993(0.672, 1.468) | 0.078 |  |
| Rhodospirillaceae | 15 | Weighted median | 0.305 | 1.092 | 0.923 | 1.292 | 1.092(0.923, 1.292) |  |  |
| Rhodospirillaceae | 15 | Simple mode | 0.116 | 1.244 | 0.964 | 1.607 | 1.244(0.964,1.607) |  |  |
| ClostridialesvadinBB60group | 12 | IVW | 0.018 | 0.865 | 0.767 | 0.975 | 0.865(0.767, 0.975) | 0.434 | 0.278 |
| ClostridialesvadinBB60group | 12 | MR Egger | 0.106 | 0.684 | 0.449 | 1.040 | 0.684(0.449, 1.040) | 0.459 |  |
| ClostridialesvadinBB60group | 12 | Weighted median | 0.041 | 0.840 | 0.711 | 0.993 | 0.840(0.711, 0.993) |  |  |
| ClostridialesvadinBB60group | 12 | Simple mode | 0.056 | 1.402 | 1.020 | 1.927 | 1.402(1.020, 1.927) |  |  |
| Defluviitaleaceae | 8 | IVW | 0.029 | 0.807 | 0.665 | 0.979 | 0.807(0.665, 0.979) | 0.479 | 0.586 |
| Defluviitaleaceae | 8 | MR Egger | 0.913 | 1.056 | 0.413 | 2.705 | 1.056(0.413, 2.705) | 0.402 |  |
| Defluviitaleaceae | 8 | Weighted median | 0.030 | 0.758 | 0.589 | 0.974 | 0.758(0.589, 0.974) |  |  |
| Defluviitaleaceae | 8 | Simple mode | 0.192 | 0.823 | 0.625 | 1.083 | 0.823(0.625, 1.083) |  |  |
| Desulfovibrionaceae | 15 | IVW | 0.035 | 1.126 | 1.008 | 1.257 | 1.126(1.008, 1.257) | 0.298 | 0.938 |
| Desulfovibrionaceae | 15 | MR Egger | 0.694 | 1.105 | 0.680 | 1.795 | 1.105(0.680, 1.795) | 0.236 |  |
| Desulfovibrionaceae | 15 | Weighted median | 0.046 | 1.163 | 1.002 | 1.348 | 1.163(1.002, 1.348) |  |  |
| Desulfovibrionaceae | 15 | Simple mode | 0.199 | 0.727 | 0.469 | 1.129 | 0.727(0.469, 1.129) |  |  |
| Coprococcus2 | 9 | IVW | 0.002 | 0.782 | 0.668 | 0.916 | 0.782(0.668, 0.916) | 0.567 | 0.739 |
| Coprococcus2 | 9 | MR Egger | 0.397 | 0.673 | 0.285 | 1.592 | 0.673(0.285, 1.592) | 0.472 |  |
| Coprococcus2 | 9 | Weighted median | 0.030 | 0.788 | 0.635 | 0.977 | 0.788(0.635, 0.977) |  |  |
| Coprococcus2 | 9 | Simple mode | 0.064 | 0.670 | 0.464 | 0.966 | 0.670(0.464, 0.966) |  |  |
| Holdemanella | 11 | IVW | 0.029 | 1.125 | 1.012 | 1.250 | 1.125(1.012 ,1.250) | 0.811 | 0.387 |
| Holdemanella | 11 | MR Egger | 0.140 | 1.282 | 0.949 | 1.731 | 1.282(0.949 ,1.731) | 0.814 |  |
| Holdemanella | 11 | Weighted median | 0.061 | 1.144 | 0.994 | 1.316 | 1.144(0.994 ,1.316) |  |  |
| Holdemanella | 11 | Simple mode | 0.123 | 1.208 | 0.970 | 1.503 | 1.208(0.970, 1.503) |  |  |
| LachnospiraceaeUCG001 | 12 | IVW | 0.024 | 1.152 | 1.019 | 1.303 | 1.152(1.019 ,1.303) | 0.622 | 0.837 |
| LachnospiraceaeUCG001 | 12 | MR Egger | 0.471 | 1.216 | 0.730 | 2.026 | 1.216(0.730 ,2.026) | 0.537 |  |
| LachnospiraceaeUCG001 | 12 | Weighted median | 0.012 | 1.230 | 1.046 | 1.446 | 1.230(1.046 ,1.446) |  |  |
| LachnospiraceaeUCG001 | 12 | Simple mode | 0.108 | 1.313 | 0.968 | 1.779 | 1.313(0.968, 1.779) |  |  |
| Senegalimassilia | 5 | IVW | 0.041 | 1.206 | 1.008 | 1.444 | 1.206(1.008 ,1.444) | 0.842 | 0.896 |
| Senegalimassilia | 5 | MR Egger | 0.719 | 1.149 | 0.576 | 2.292 | 1.149 (0.576 ,2.292) | 0.708 |  |
| Senegalimassilia | 5 | Weighted median | 0.157 | 1.179 | 0.939 | 1.481 | 1.179(0.939 ,1.481) |  |  |
| Senegalimassilia | 5 | Simple mode | 0.253 | 1.229 | 0.908 | 1.664 | 1.229(0.908, 1.664) |  |  |
| Terrisporobacter | 5 | IVW | 0.021 | 1.222 | 1.031 | 1.447 | 1.222(1.031, 1.447) | 0.822 | 0.547 |
| Terrisporobacter | 5 | MR Egger | 0.254 | 1.443 | 0.866 | 2.406 | 1.443(0.866, 2.406) | 0.785 |  |
| Terrisporobacter | 5 | Weighted median | 0.025 | 1.269 | 1.031 | 1.563 | 1.269(1.031, 1.563) |  |  |
| Terrisporobacter | 5 | Simple mode | 0.114 | 1.326 | 1.008 | 1.744 | 1.326(1.008, 1.744) |  |  |
